# Supplementary material for: Joint single-cell multiomic analysis in Wnt3a induced asymmetric stem cell division
Source: Nat Commun. 2021 Oct 12;12:5941. doi: 10.1038/s41467-021-26203-0 (PMC8511096; doi:10.1038/s41467-021-26203-0)
Supplement: Supplementary file 1 — Supplementary Information [file 41467_2021_26203_MOESM1_ESM.pdf]

Joint single-cell multiomic analysis in Wnt3a induced asymmetric stem cell division

Zhongxing Sun[1, #], Yin Tang[1, #], Yanjun Zhang[1, #], Yuan Fang[1, #], Junqi Jia[1], Weiwu Zeng[1], and Dong Fang[1, 2, *]

[1] Zhejiang Provincial Key Laboratory for Cancer Molecular Cell Biology, Life Sciences Institute, Zhejiang University, Hangzhou, Zhejiang 310058, China.

[2] Department of Medical Oncology, Key Laboratory of Cancer Prevention and Intervention, Ministry of Education, The Second Affiliated Hospital, Zhejiang University School of Medicine, Hangzhou, Zhejiang 310058, China.

[#] These authors contribute equally

[*] Corresponding author: Dong Fang, Email: dfang@zju.edu.cn

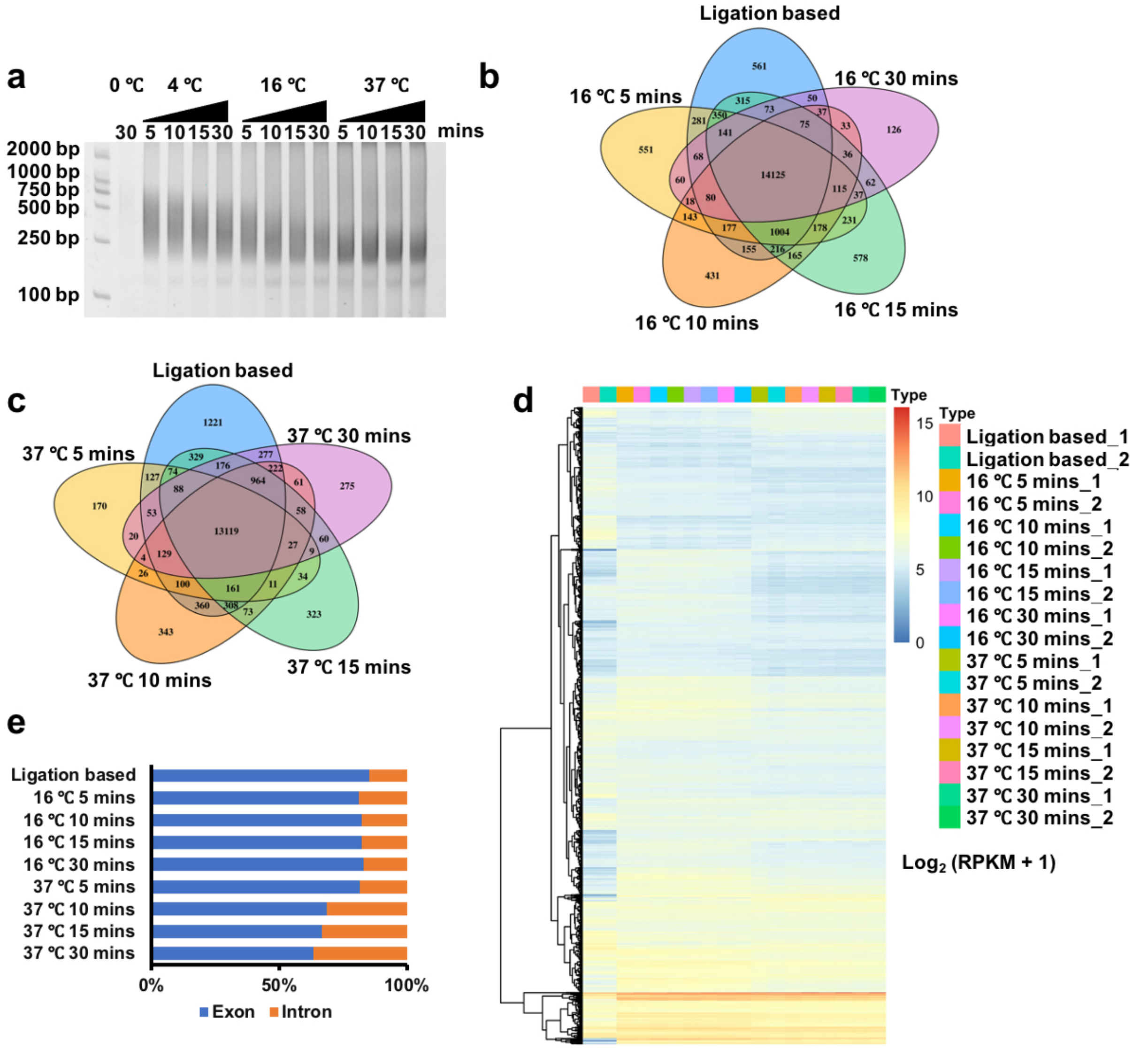

Supplementary Figure S1. The impacts of tagmentation time and temperature on the gene expression libraries are analyzed.

(**a**) PCR fragments of tagmentated cDNA at the indicated time and temperature were shown by agarose gel. cDNAs were tagmentated at the indicated time and temperature, PCR amplified, and analyzed by TBE gel.

(**b**) Venn diagram showing the overlap of detected genes among different libraries. The gene expression libraries were constructed from tagmentation under 16 ℃ for the indicated time. Ligation-based gene expression libraries were used as control.

(**c**) Venn diagram showing the overlap of identified genes among different libraries. The gene

expression libraries were constructed from tagmentation under 37 °C for the indicated time.
(**d**) Gene expression heatmap showing similar expression levels of detected genes among different libraries. Genes with expression levels ranked as top 2000 were plotted. Libraries were constructed from tagmentation under the indicated time and temperature. RPKM, Reads Per Kilobase per Million mapped reads.
(**e**) Bar graph showing the ratio of detected reads at exons and introns. The libraries were constructed from the indicated tagmentation time and temperature. The number of reads mapped to exons or introns was calculated by Homer.
Source data are provided as a Source Data file.

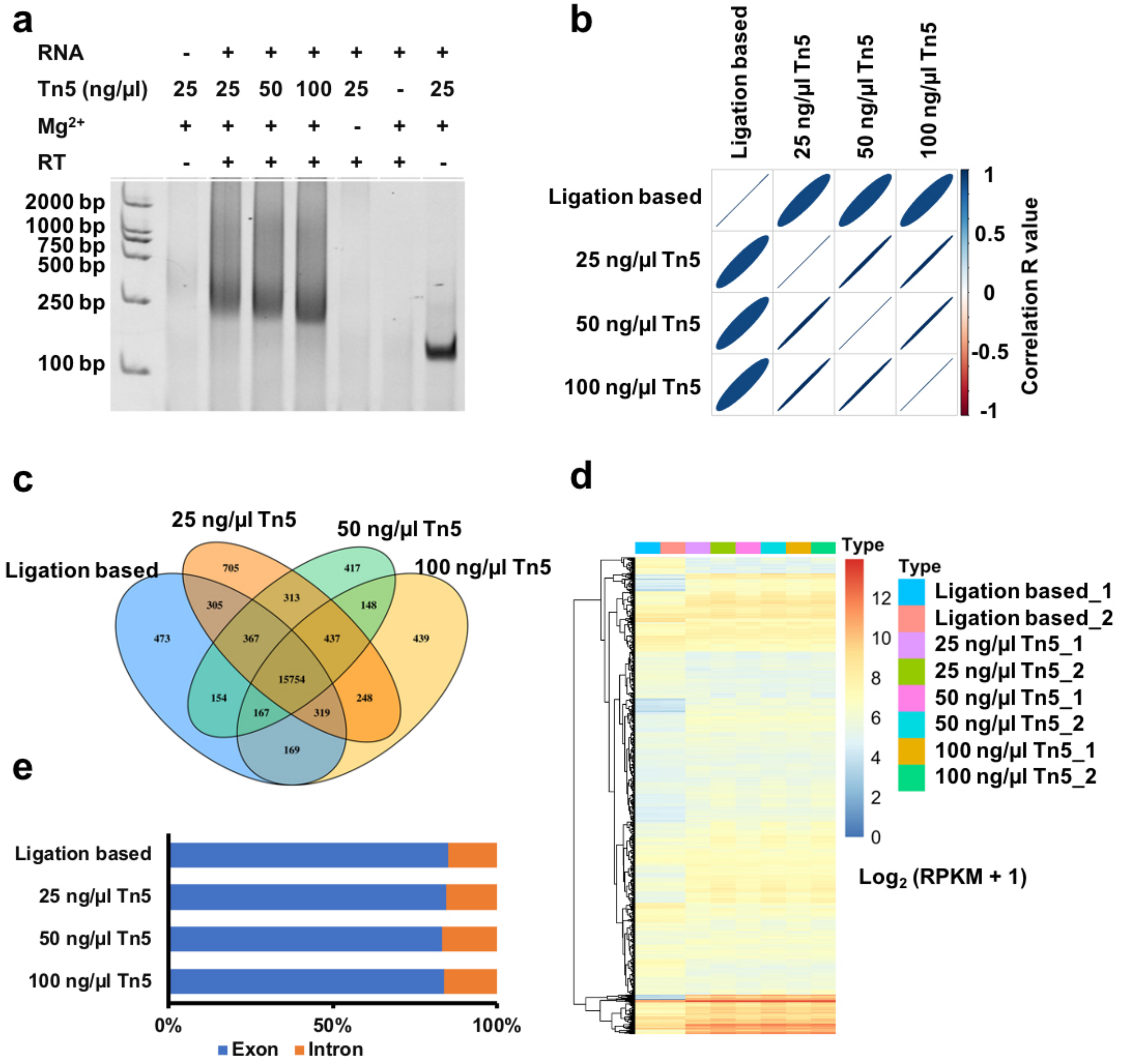

Supplementary Figure S2. Different amounts of Tn5 don't affect the constructed gene expression libraries.

(**a**) PCR fragments of tagmentated cDNA were shown by agarose gel. cDNAs were tagmentated at 37 °C for 5 mins with the indicated amount of Tn5, PCR amplified, and analyzed by TBE gel. RT, reverse transcription.

(**b**) Correlations among gene expression libraries constructed from the increased amounts of Tn5. The Pearson correlation coefficient was calculated.

(**c**) Venn diagram showing similar detected genes among gene expression libraries constructed

from increased amounts of Tn5.

(**d**) Gene expression heatmap showing similar expression levels of detected genes with the increased amount of Tn5. Genes with expression levels ranked as top 2000 were plotted. Libraries were constructed from tagmentation under the indicated amount of Tn5.

(**e**) Bar graph showing similar ratios of detected reads at exons and introns. The gene expression libraries were constructed from the indicated amount of Tn5. The number of reads mapped to exons or introns was calculated by Homer.

Source data are provided as a Source Data file.

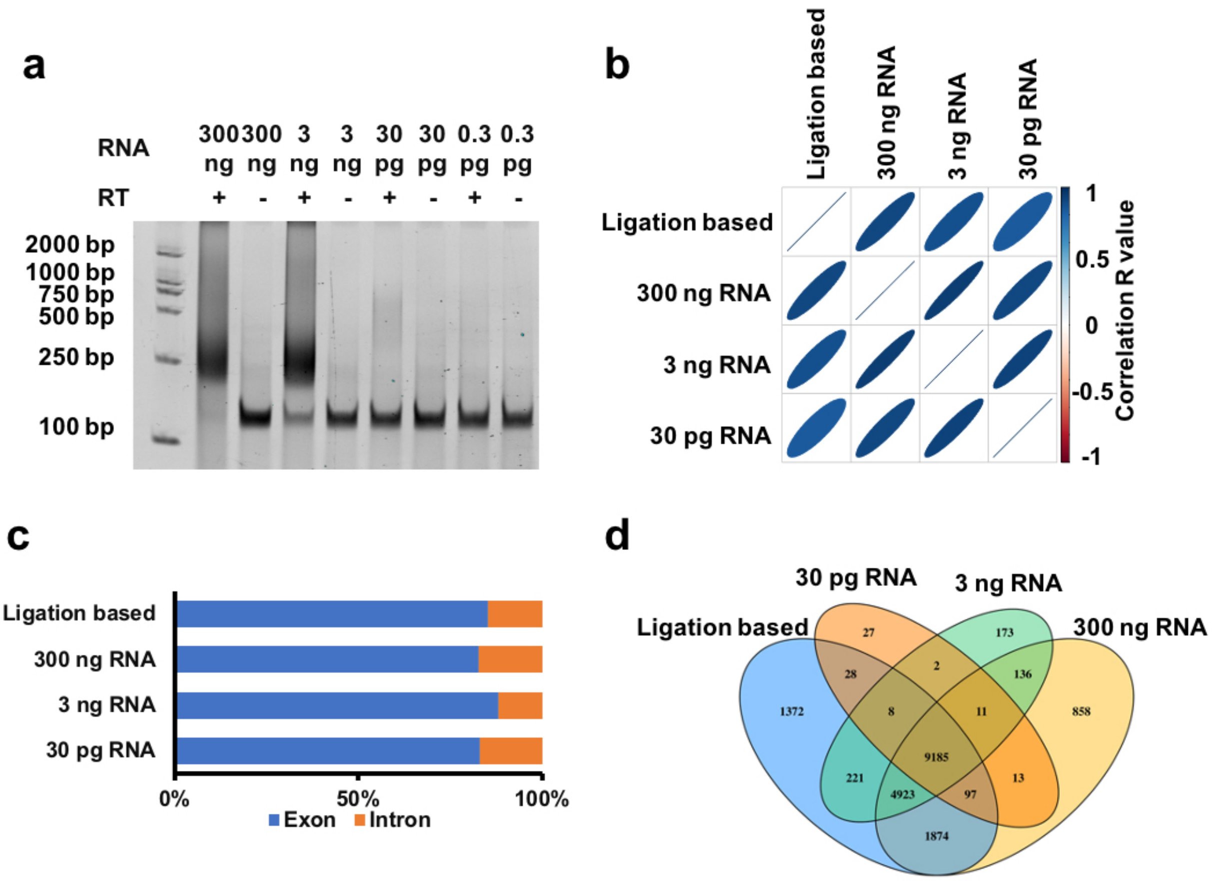

Supplementary Figure S3. Total RNA as low as 30 pg is enough for the library construction.

(**a**) PCR fragments of tagmentated cDNA were shown by TBE gel. The indicated amount of total RNA was used as starting material. cDNAs were tagmentated at 37 °C for 5 mins, PCR amplified, and analyzed by agarose gel. RT, reverse transcription.

(**b**) The Pearson correlations among gene expression libraries constructed from the indicated amount of total RNA.

(**c**) Bar graph showing similar ratios of detected reads at exons and introns in the libraries constructed from the indicated amount of total RNA.

(**d**) Venn diagram showing similar detected genes among libraries constructed from the indicated amount of total RNA.

Source data are provided as a Source Data file.

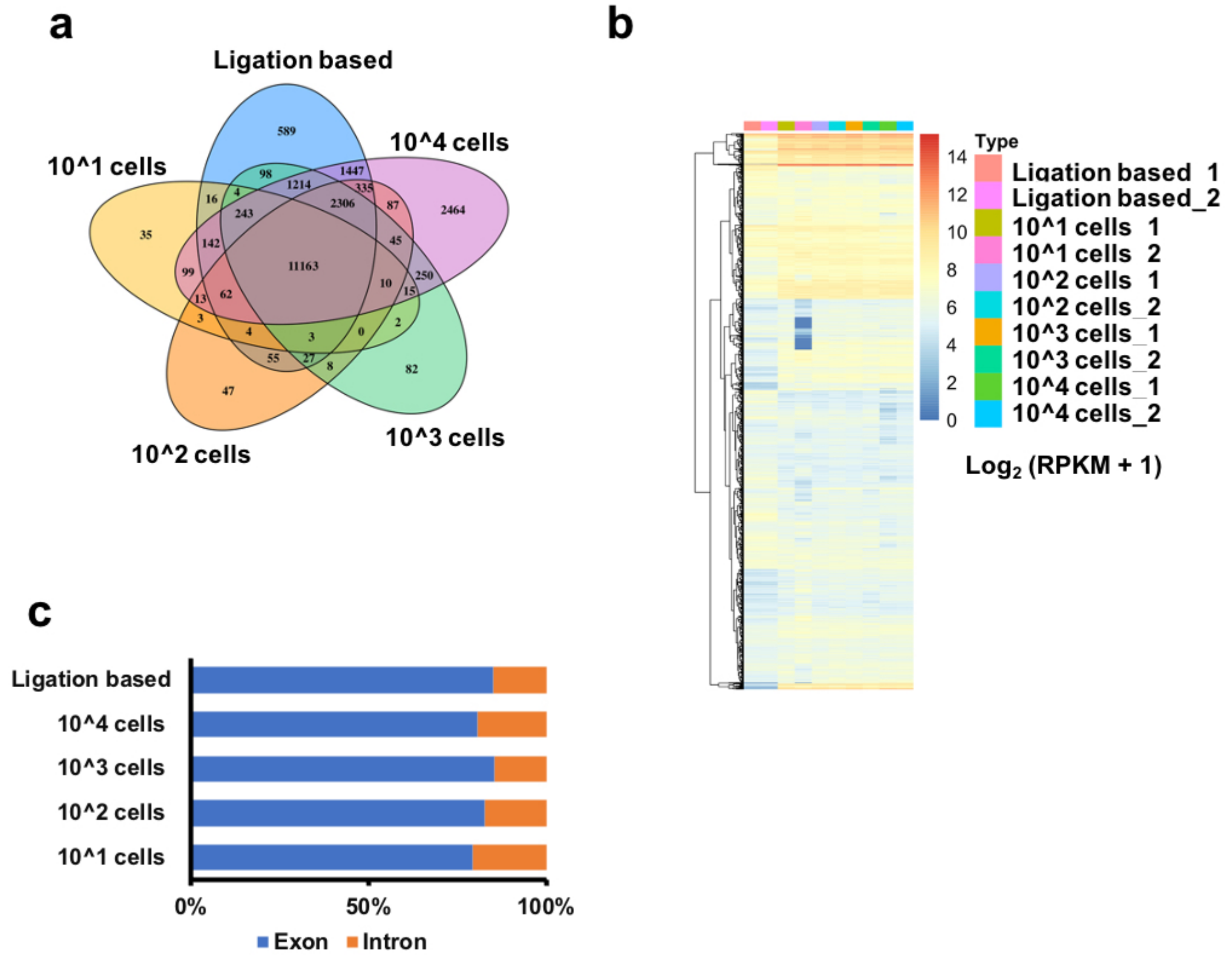

Supplementary Figure S4. The low number of cells could be used for gene expression library construction in SET-seq.

(**a**) Venn diagram showing detected genes among libraries constructed from the indicated number of cells. 10 to 10,000 cells were used as the starting material for the gene expression libraries construction of SET-seq.

(**b**) Gene expression heatmap showing expression levels of detected genes with the indicated number of cells. Genes with expression levels ranked as top 2000 were plotted. Genes detected from 10 cells were less than the other numbers of cells tested.

(**c**) Bar graph showing similar ratios of detected reads at exons and introns in the libraries constructed from the indicated number of cells.

Source data are provided as a Source Data file.

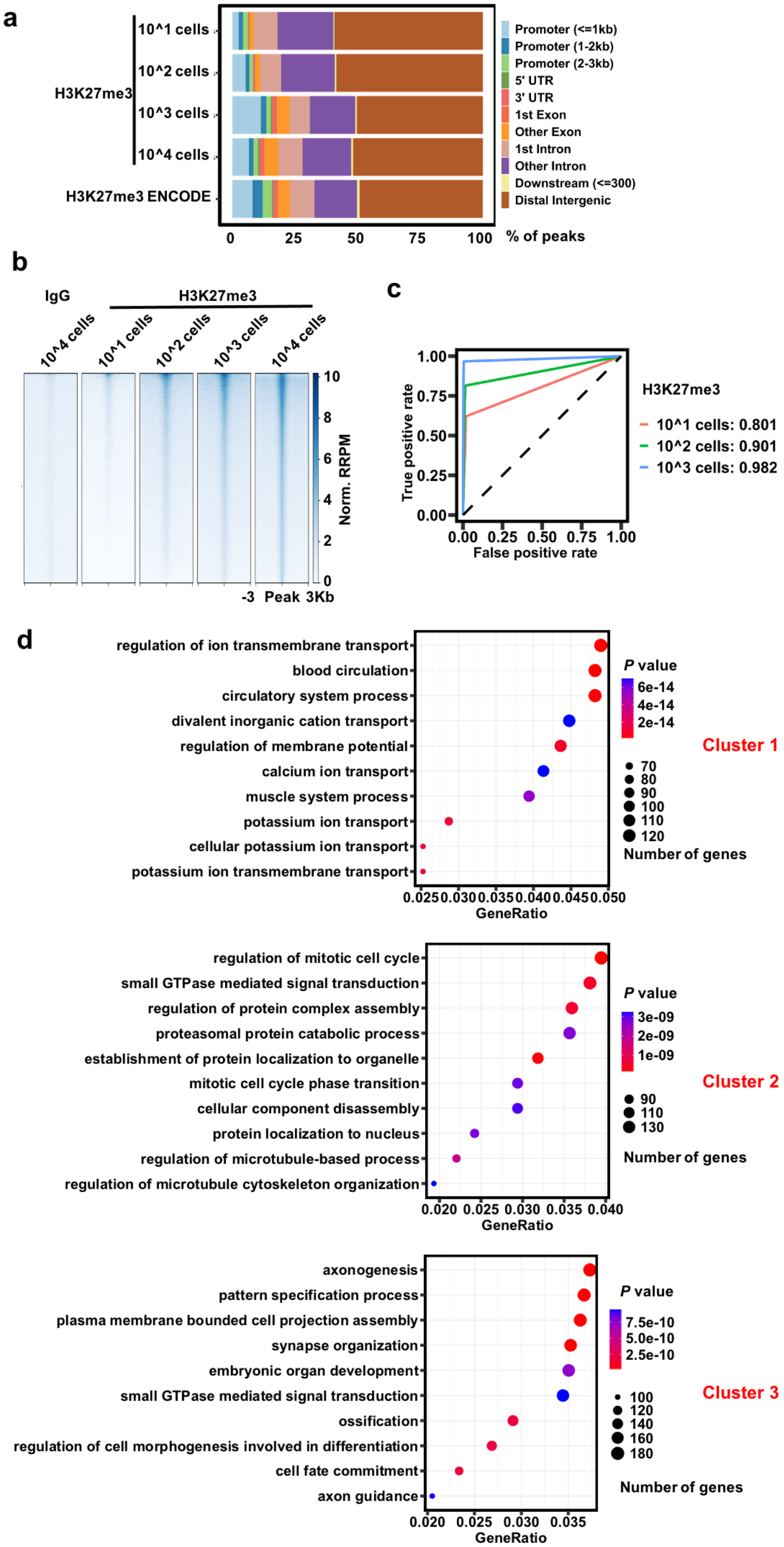

Supplementary Figure S5. SET-seq can be used for library preparation from 10 to 10,000

cells.

(**a**) The percentages of H3K27me3 peaks in the genome. H3K27me3 ChIP-seq and its input were downloaded from ENCODE data ENCSR059MBO [https://www.encodeproject.org/experiments/ENCSR059MBO/] and ENCSR326ULS [https://www.encodeproject.org/experiments/ENCSR326ULS/] as reference. Two repeats of each SET-seq result were merged.

(**b**) Heatmaps illustrating H3K27me3 levels at peaks in the data of 10,000 cells. H3K27me3 levels from 3 Kb upstream to 3 Kb downstream of the peak regions (in rows) are shown on a per-peak basis (in columns). Norm. RRPM, Normalized Reference-adjusted Reads Per Million.

(**c**) Receiver operating characteristic curve (ROC) showing a high AUC (Area under curve) value in H3K27me3 SET-seq libraries constructed from 10 to 1,000 cells.

(**d**) The scatterplot shows the gene ontology (GO) analysis of genes clustered as in Figure 1d using 10,000 cells. The color indicated the *P* value. The dot size was scaled according to the ratio of genes in the GO terms.

Source data are provided as a Source Data file.

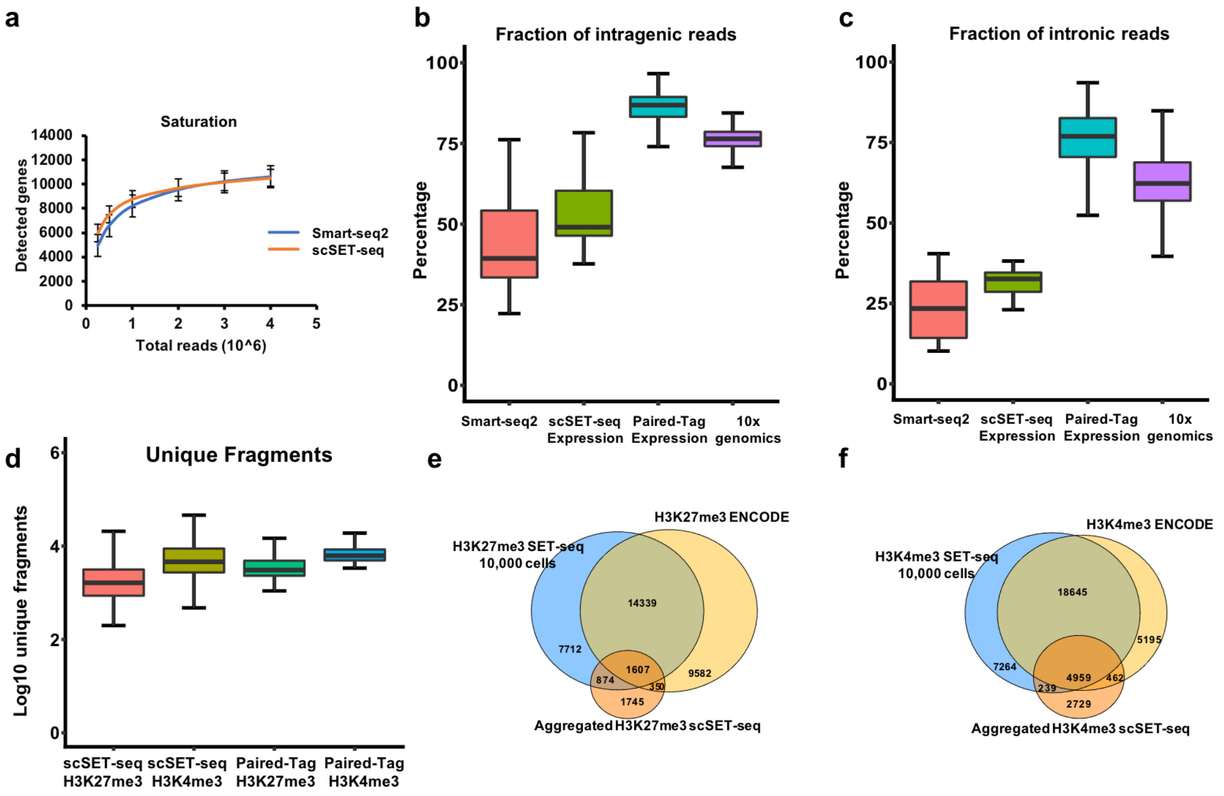

Supplementary Figure S6. The quality of reads in scSET was similar to Smart-Seq2 and Paired-Tag.

(**a**) scSET and Smart-seq2 shared a plateau of saturation at around 1 to 2 million total reads. 48 cells from SMART-seq2 data, which were downloaded from GSE151334 [https://www.ncbi.nlm.nih.gov/geo/query/acc.cgi], were randomly selected. Saturation curves were constructed with randomly gradient. n = 4M, 3M, 2M,1M, 0.5M, 0.25M reads from total reads. Data were mean ± SD.

(**b** and **c**) Boxplots showing the percentages of mapped reads in intragenic (b) and intronic (c) regions. Paired-Tag and 10x genomics data were adapted from previous reports[1]. The boxes were drawn from lower quartile (Q1) to upper quartile (Q3) with the middle line denoting the median, and whiskers with maximum 1.5 IQR (interquartile range). n = 48 (Smart-seq2), 48 (scSET-seq Expression), 27,663 (Paired-Tag Expression), and 5,828 (10x genomics).

(**d**) Boxplots showing the number of unique fragments detected. Unique fragments in scSET-seq and Paired-Tag were deduplicated by GATK and reachtools [https://github.com/cxzhu/Paired-Tag], respectively, to fit the different sequencing library structures. The boxes were drawn from lower

quartile (Q1) to upper quartile (Q3) with the middle line denoting the median, and whiskers with maximum 1.5 IQR (interquartile range). n = 400 Cells (top 400 total sequencing reads) in each library.

(**e** and **f**) Venn diagrams showing the overlaps of H3K27me3 (e) and H3K4me3 (f) SET-seq peaks from 10,000 cells, ENCODE data peaks, and merged scSET-seq peaks, respectively. Fisher's exact statistical tests were done for the overlaps and *P* values were less than 0.01 between every two overlaps.

Source data are provided as a Source Data file.

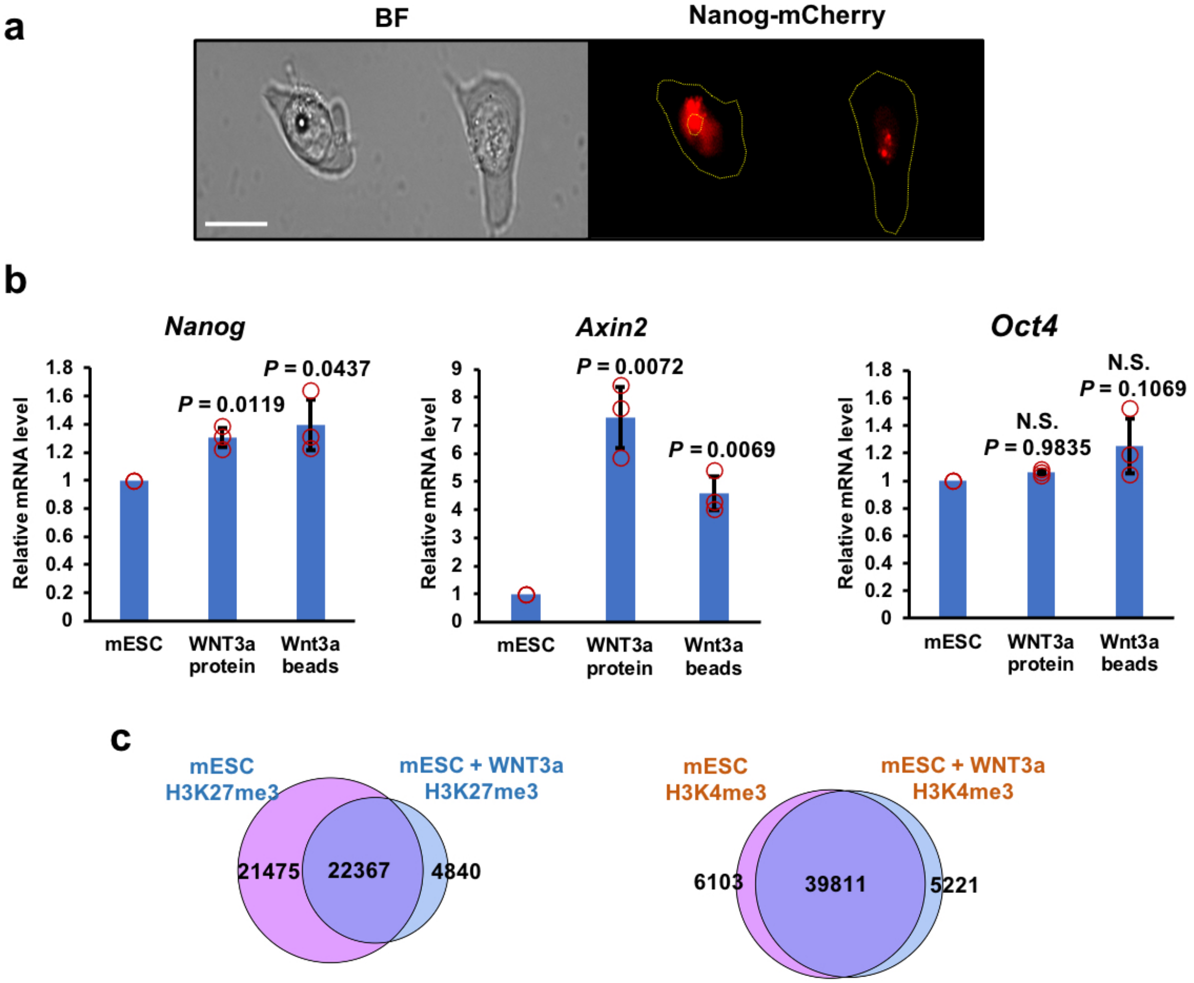

Supplementary Figure S7. Wnt3a beads induce asymmetric cell division.

(**a**) Representative image showing the asymmetric divided Nanog-mCherry mESCs. The Wnt3a bead and cells were marked in the immunofluorescence image. A higher mCherry signal was detected in the bead-proximal cell. BF, bright field. Scale bar, 15 μm.

(**b**) RT-PCR results showing the successful induction of higher *Nanog* and *Axin2* expressions with Wnt3a beads. Cells with WNT3a protein were used as a positive control for the activity of WNT3a. Large amounts of Wnt3a beads were used to attach most cells with localized Wnt3a signals. Results represented the mean ± SD (n = 3 biologically independent replications). *P* values were calculated by Student's t-test, two-sided.

(**c**) Venn diagrams showing the overlap of H3K27me3 and H3K4me3 SET-seq peaks with or without WNT3a treatment, respectively.

Source data are provided as a Source Data file.

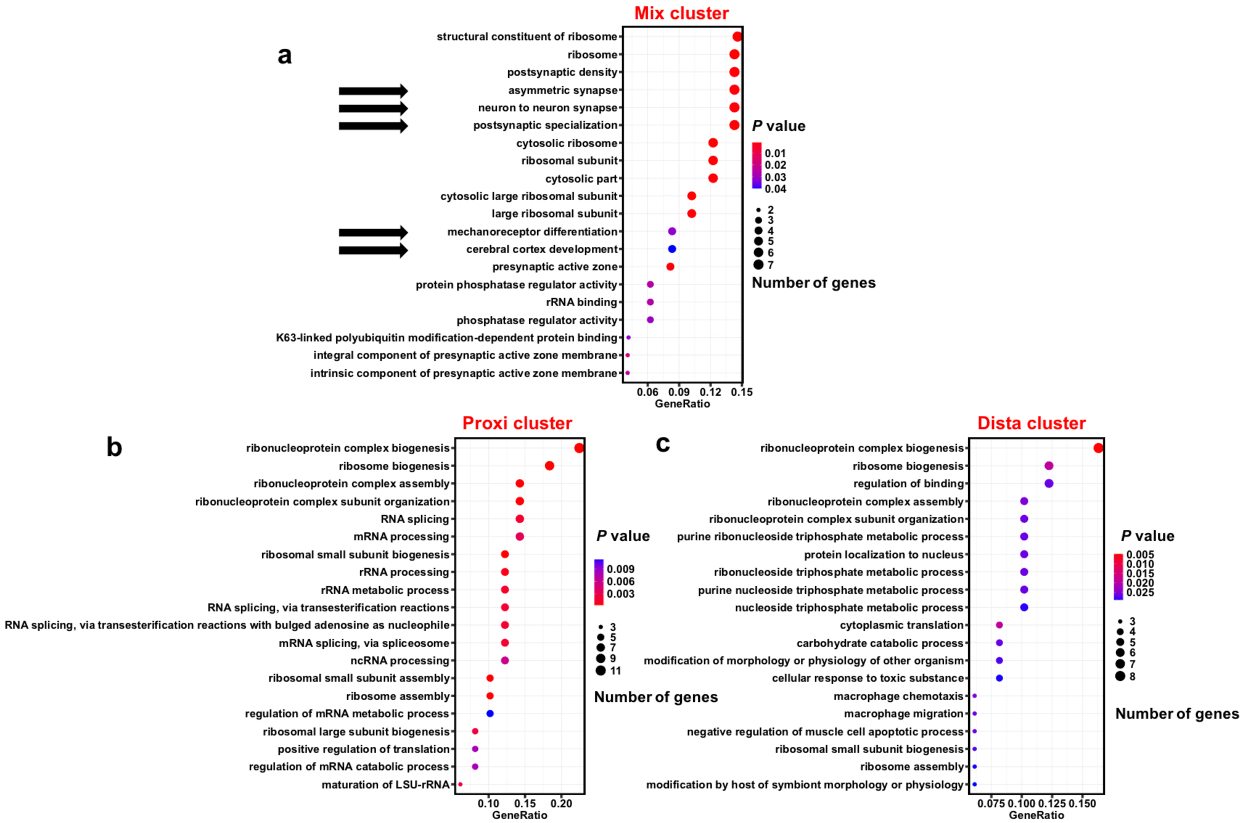

Supplementary Figure S8. GO terms of cluster marker genes enrichments.

(**a**) The scatterplot shows the gene ontology (GO) analysis of all marker genes of the Mix cluster. The color indicated the *P* value. The dot size was scaled according to the ratio of genes in the GO terms. Arrows indicated the GO terms associated with neuron interaction and differentiation.

(**b**) Same as in (a), except marker genes of the Proxi cluster were analyzed.

(**c**) Same as in (a), except marker genes of the Dista cluster were analyzed.

Source data are provided as a Source Data file.

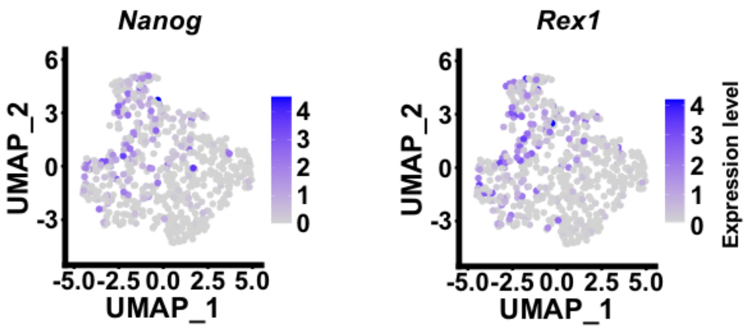

Supplementary Figure S9. Nanog and Rex1 are upregulated in cells of the Proxi cluster.

Gene expression levels of *Nanog* and *Rex1* were plotted in individual cells. Cells were clusters by the merged expression profiles of H3K27me3 and H3K4me3 scSET-seq.

Source data are provided as a Source Data file.

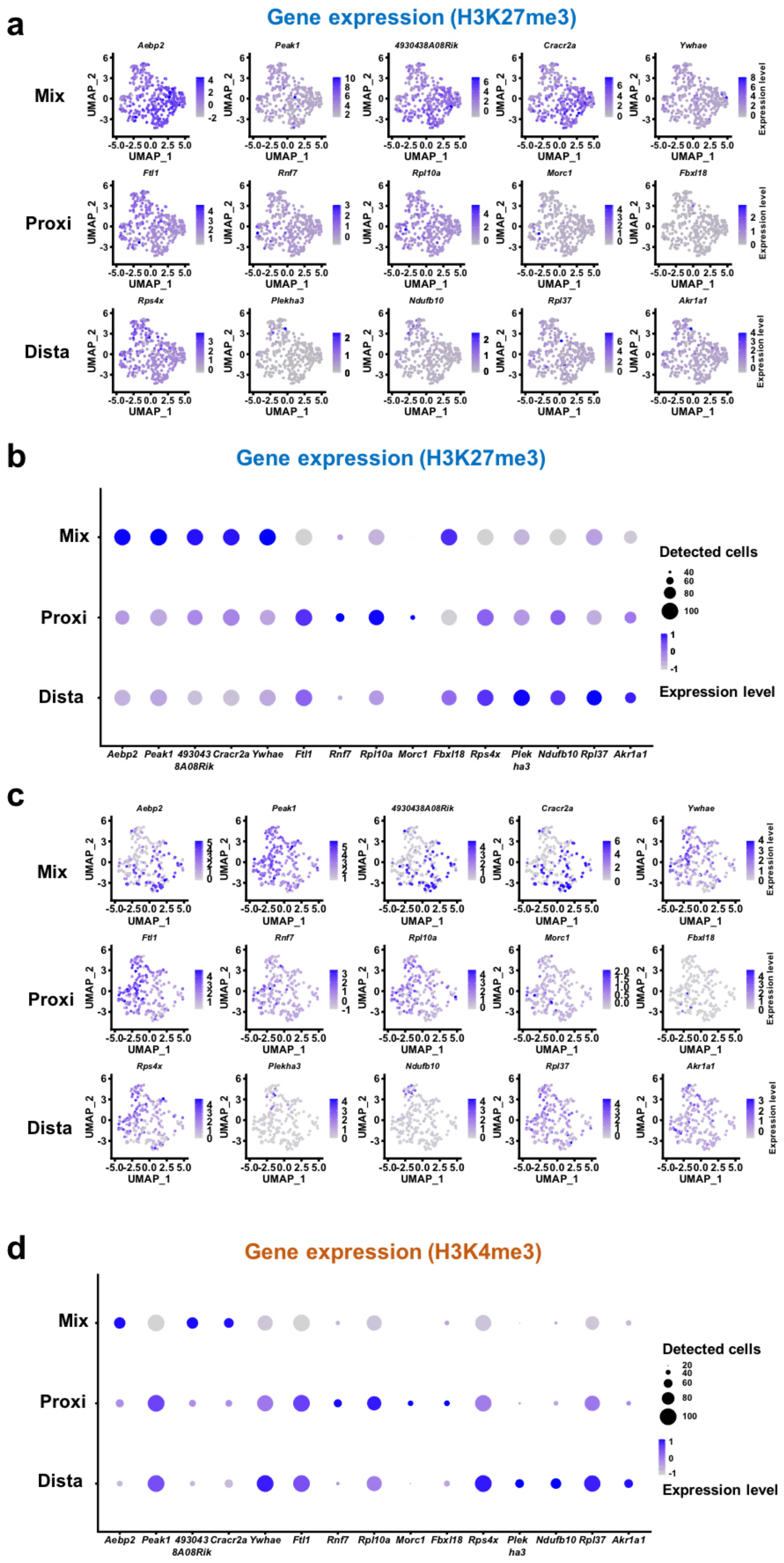

Supplementary Figure S10. Marker genes are elevated in H3K27me3 and H3K4me3 scSET-

seq.

(**a**) Expression levels of the top 5 marker genes, which were ranked by Log_2(*FoldChange*), were plotted in individual cells of H3K27me3 scSET-seq.

(**b**) Feature plot showing the top 5 marker genes, which were ranked by Log_2(*FoldChange*), across the Mix, Proxi, and Dista clusters in H3K27me3 scSET-seq.

(**c**) Same as in (a), except H3K4me3 scSET-seq was analyzed.

(**d**) Same as in (b), except H3K4me3 scSET-seq was analyzed.

Source data are provided as a Source Data file.

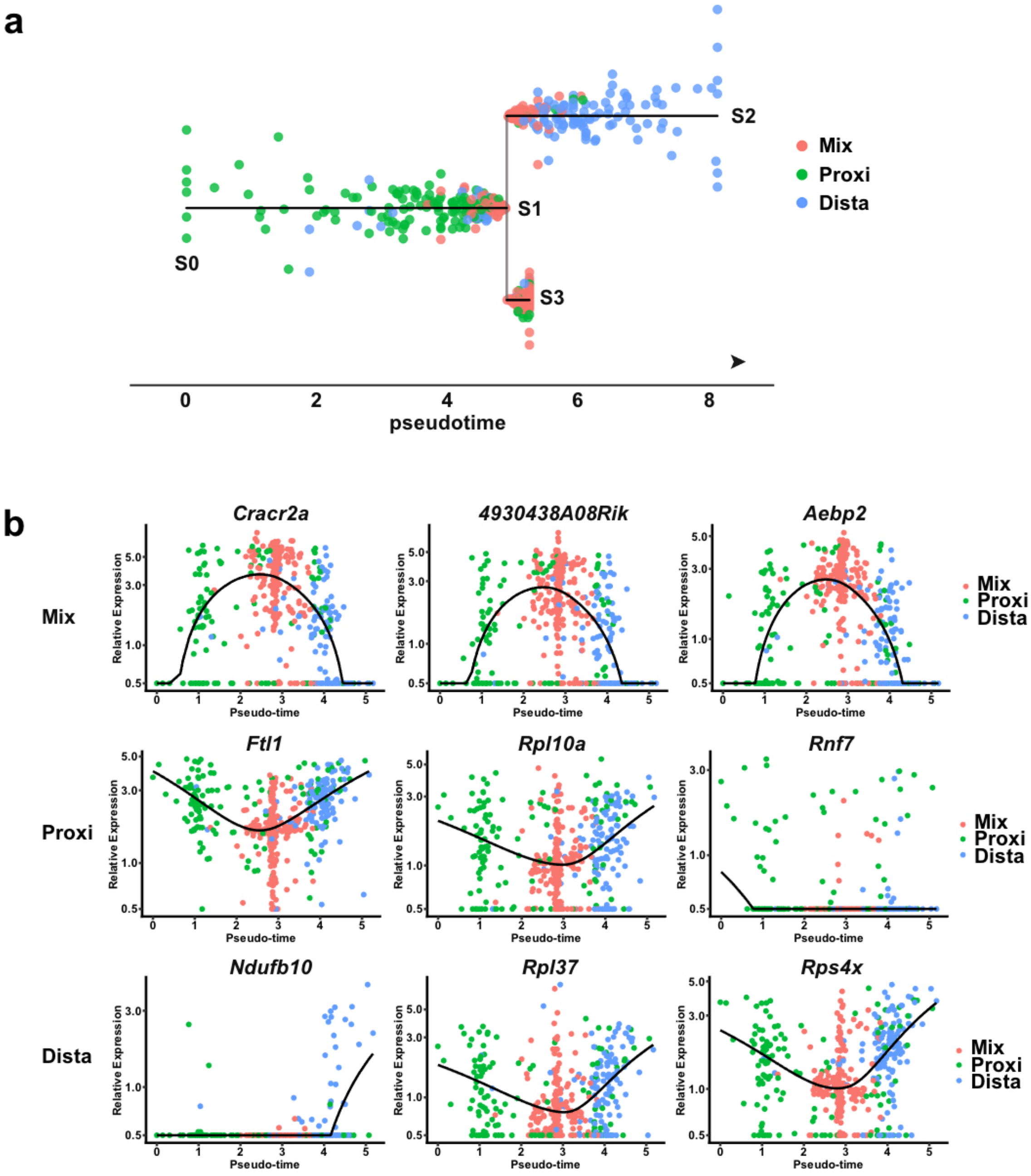

Supplementary Figure S11. The single cells were assigned into different trajectories.

(**a**) Subway map plot of the assignment of single cells. Each cell is colored by their clusters. S1-S4 produced by STREAM, indicated the cell trajectories.

(**b**) The dynamics of the expression levels of marker genes across the pseudotemporal trajectories. Three representative marker genes from each cluster were selected to be plotted.

Source data are provided as a Source Data file.

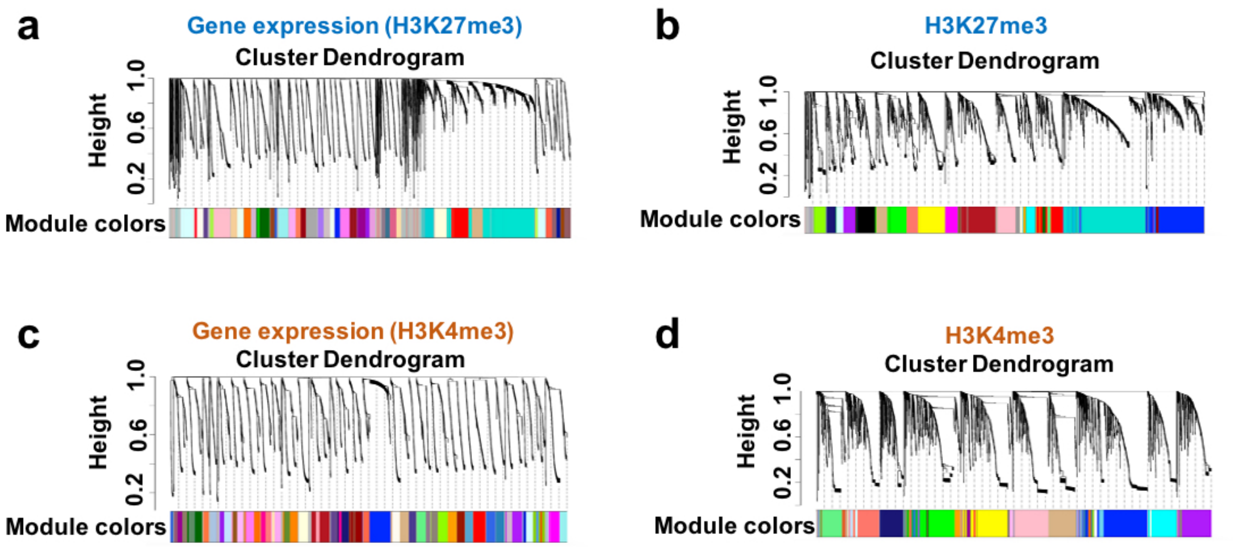

Supplementary Figure S12. H3K27me3 and H3K4me3 scSET-seq are clustered into WCGNA modules.

(**a**) Gene expression profiles of H3K27me3 scSET-seq were clustered into WCGNA modules. Genes with detected transcriptional and epigenomic signals were selected for the generation of modules.

(**b**) H3K27me3 profiles of H3K27me3 scSET-seq were clustered into WCGNA modules. Genes with detected transcriptional and epigenomic signals were selected for the generation of modules.

(**c**) Same as in (a), except H3K4me3 scSET-seq was analyzed.

(**d**) Same as in (b), except H3K4me3 scSET-seq was analyzed.

Source data are provided as a Source Data file.

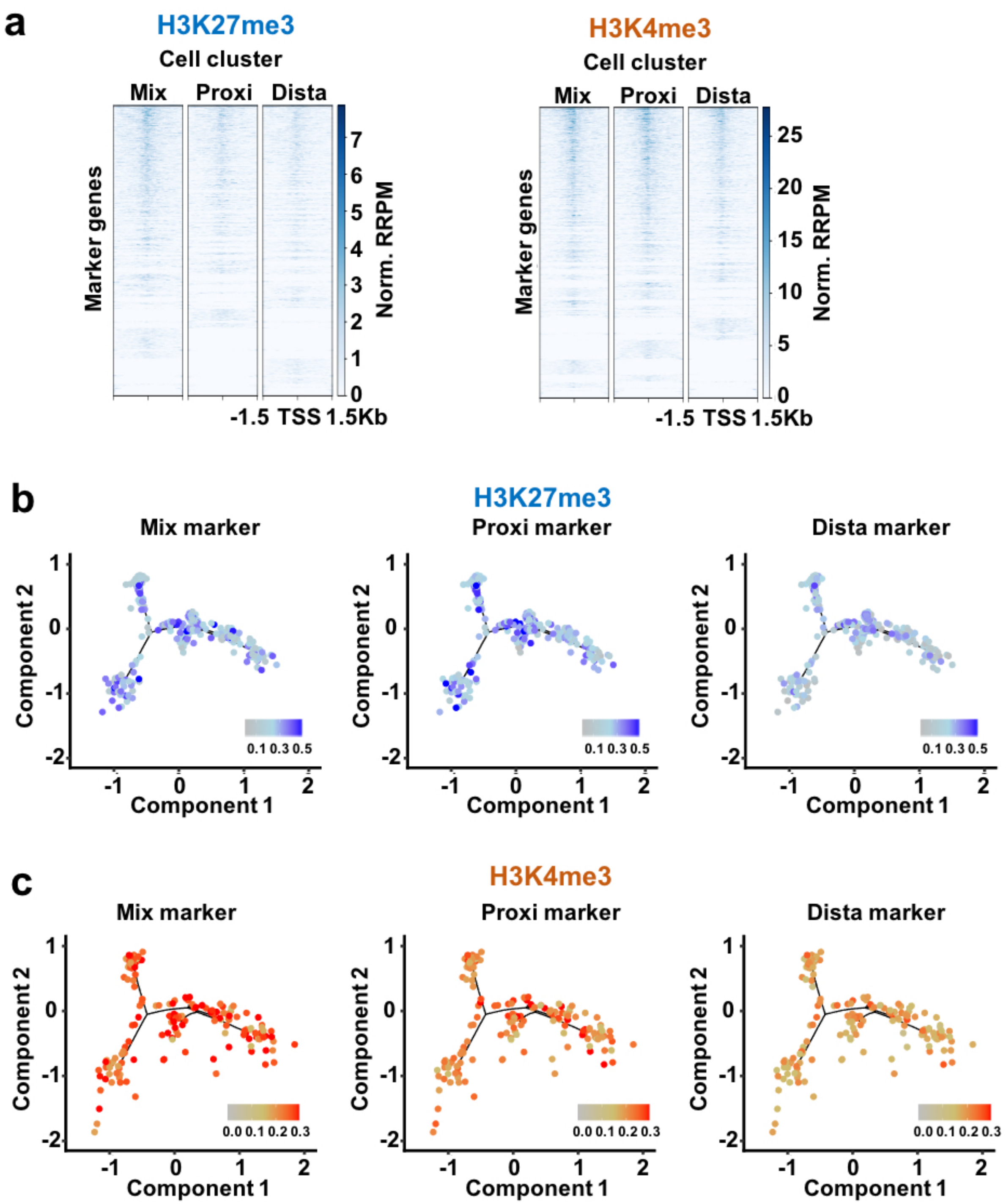

Supplementary Figure S13. H3K27me3 is elevated at marker genes in cells of the Mix cluster. (**a**) Heatmaps illustrating H3K27me3 and H3K4me3 levels at marker genes in different clusters of cells, respectively. H3K27me3 and H3K4me3 levels from 1.5 Kb upstream to 1.5 Kb downstream of the TSS (in rows) are shown on a per-gene basis (in columns), respectively. SET-seq results of

the cells in each cluster were aggregated for analysis. Norm. RRPM, Normalized Reference-adjusted Reads Per Million.

(**b** and **c**) H3K27me3 (b) and H3K4me3 (c) signal at marker genes of different clusters across the pseudotemporal trajectories. H3K27me3 and H3K4me3 signals of cluster marker genes in each cell were aggregated during the plotting. Colors indicated the signal levels.

Source data are provided as a Source Data file.

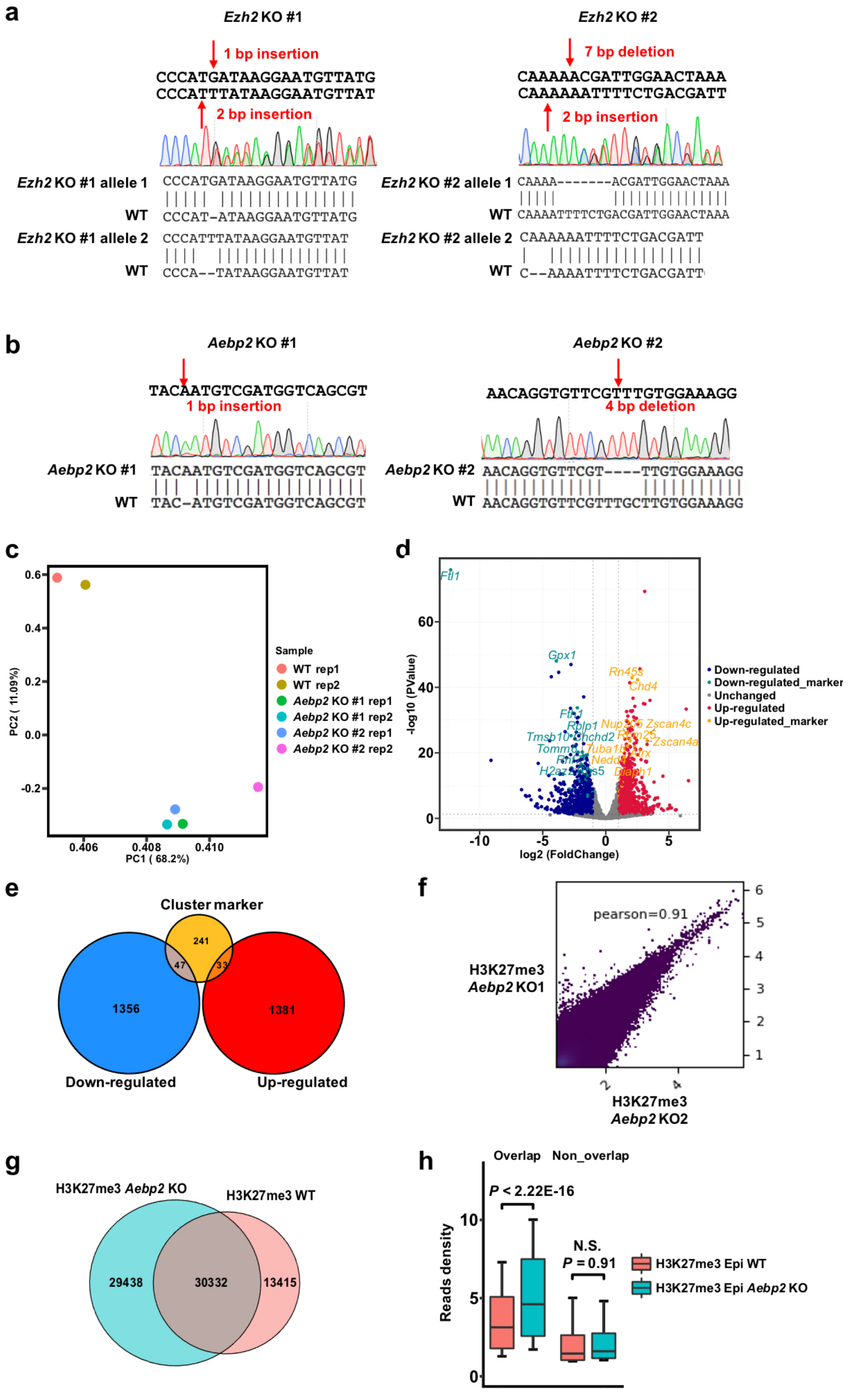

Supplementary Figure S14. Aebp2 and Ezh2 are knocked out in individual cell lines.

(**a**) Two alleles of *Ezh2* were mutated differently in two sgRNA knockout clones. The Sanger sequencing results of the *Ezh2* gene locus, which was amplified from genomic DNA, were shown. The red arrow indicated the mutation sites. One allele in the *Ezh2* knockout #1 cell line contained 1 bp insertion, and the other allele represented 2 bp insertion, indicating two alleles were knocked out. One allele in the *Ezh2* knockout #2 cell line bared a 7 bp deletion, and the other allele in the *Ezh2* knockout #2 cell line exhibited 2 bp insertion, showing two alleles were knocked out.
(**b**) Two alleles of *Aebp2* were mutated in knockout clones. The Sanger sequencing results of the *Aebp2* gene locus were shown. 1 bp insertion and 4 bp deletion were detected at both alleles in *Aebp2* knockout #1 and #2 clones, respectively.
(**c**) The principal component analysis (PCA) plot of the gene expression data from wild-type (WT), and *Aebp2* KO mESCs. Two replicates of each cell line were presented.
(**d**) Gene expression levels in wild-type and *Aebp2* KO mESCs. To get the reproducible results, RNA sequencing data from two replicates of each cell line were merged and two *Aebp2* KO cell lines were further merged. Red dots, the up-regulated genes with $\log_2(FoldChange) > 1$ and P value less than 0.05 in *Aebp2* KO mESCs. Blue dots, the down-regulated genes with $\log_2(FoldChange) < -1$ and P value less than 0.05 in *Aebp2* KO mESCs. Yellow and cyan dots, the up-regulated and down-regulated cluster marker genes, respectively. Top 10 changed marker genes were shown.
(**e**) Venn diagram illustrating the overlap of changed genes and cluster marker genes.
(**f**) Scatter plot showing the correlations between two *Aebp2* KO cell lines. A 1 Kb sliding window across the whole genome was used to calculate the Pearson product moment correlation.
(**g**) Venn diagram illustrating the overlap of H3K27me3 peaks in WT and *Aebp2* KO cells.
(**h**) The enrichments of H3K27me3 at H3K27me peaks that were overlapped or nonoverlapped with Aebp2 peaks. The reads density of H3K27me3 was calculated for each peak. *P* values were calculated by Student's t-test, two-sided. The boxes were drawn from lower quartile (Q1) to upper quartile (Q3) with the middle line denoting the median, and whiskers with maximum 1.5 IQR (interquartile range). n = 1000 (Overlap) and 1000 (Non_overlap) H3K27me3 bins.
Source data are provided as a Source Data file.

Supplementary Table 1. Correlations between two replicates of gene expression sequencing results. The correlation of gene expression repeats was calculated by the coefficient of determination (R^2) of their shared genes' RPKM values.

Samples	Correlation
H3K27me3 SET-seq 10^2 cells_Exp	0.93
H3K27me3 SET-seq 10^3 cells_Exp	0.97
H3K27me3 SET-seq 10^4 cells_Exp	0.96
H3K4me3 SET-seq 10^2 cells_Exp	0.90
H3K4me3 SET-seq 10^3 cells_Exp	0.98
H3K4me3 SET-seq 10^4 cells_Exp	0.99
Ligation based RNA seq	0.99
vitro-16℃-5mins	0.98
vitro-16℃-10mins	0.95
vitro-16℃-15mins	0.95
vitro-16℃-30mins	0.92
vitro-37℃-5mins	0.86
vitro-37℃-10mins	0.96
vitro-37℃-15mins	0.71
vitro-37℃-30mins	0.71
vitro-RNA-300ng	0.72
vitro-RNA-3ng	0.75
vitro-RNA-30pg	0.99
vitro-Tn5-25ng/µl	0.74
vitro-Tn5-50ng/µl	0.77
vitro-Tn5-100ng/µl	0.74
SET-seq 10 cells_Exp	0.86
SET-seq 100 cells_Exp	0.89
SET-seq 1,000 cells_Exp	0.96
SET-seq 10,000 cells_Exp	0.99
H3K27me3_Exp_Aebp2_KO1	1
H3K27me3_Exp_Aebp2_KO2	1

Supplementary Table 2. Correlations between two replicates of epigenomic sequencing results. A 1 Kb sliding window across the whole genome was used to calculate the Pearson product moment correlation for epigenomic sequencing.

Samples	Correlation
H3K27me3 SET-seq 10^2 cells_Epi	0.64
H3K27me3 SET-seq 10^3 cells_Epi	0.85
H3K27me3 SET-seq 10^4 cells_Epi	0.92
H3K4me3 SET-seq 10^2 cells_Epi	0.71
H3K4me3 SET-seq 10^3 cells_Epi	0.72
H3K4me3 SET-seq 10^4 cells_Epi	0.81
WNT3a H3K27me3 SET-seq 10^4 cells_Epi	0.95
WNT3a H3K4me3 SET-seq 10^4 cells_Epi	0.96
H3K27me3 SET-seq 10 cells_Epi_1	0.33
H3K27me3 SET-seq 100 cells_Epi_1	0.43
H3K27me3 SET-seq 1000 cells_Epi_1	0.55
H3K27me3 SET-seq 10000 cells_Epi_1	0.69
H3K27me3 SET-seq 10 cells_Epi_2	0.32
H3K27me3 SET-seq 100 cells_Epi_2	0.36
H3K27me3 SET-seq 1000 cells_Epi_2	0.45
H3K27me3 SET-seq 10000 cells_Epi_2	0.60
IgG SET-seq 10000 cells_Epi	0.88
H3K27me3_Epi_Aebp2_KO1	0.95
H3K27me3_Epi_Aebp2_KO2	0.92

Reference

1. Zhu, C. *et al.* Joint profiling of histone modifications and transcriptome in single cells from mouse brain. *Nat Methods* (2021).
