# Supplementary material for: Joint single-cell multiomic analysis in Wnt3a induced asymmetric stem cell division
Source: Nat Commun. 2021 Oct 12;12:5941. doi: 10.1038/s41467-021-26203-0 (PMC8511096; doi:10.1038/s41467-021-26203-0)
Supplement: Supplementary file 3 — Description of Additional Supplementary Files [file 41467_2021_26203_MOESM3_ESM.docx]

**Description of Additional Supplementary Files**

Title: Supplementary Movie 1

Description: The dividing process of beads connected cells.

Title: Supplementary Dataset 1

Description: The metadata of epigenetic scSET-seq data.

Title: Supplementary Dataset 2

Description: Index list for the scSET-seq.

Title: Supplementary Dataset 3

Description: The metadata of scSET-seq data during wnt3a beads inducted cell division.

Title: Supplementary Dataset 4

Description: Exact *P* values provided in Fig. 3a.

Title: Supplementary Dataset 5

Description: The correlations and *P* values between epigenetic and transcriptional as calculated by WGCNA.

Title: Supplementary Dataset 6

Description: The primer and barcode sequences used in SET-seq experiments.

Title: Supplementary Dataset 7

Description: The number of total reads and unique mapped reads.

Title: Supplementary Dataset 8

Description: Peak qualities of bulk-seq.
