# Supplementary material for: Joint single-cell multiomic analysis in Wnt3a induced asymmetric stem cell division
Source: Nat Commun. 2021 Oct 12;12:5941. doi: 10.1038/s41467-021-26203-0 (PMC8511096; doi:10.1038/s41467-021-26203-0)
Supplement: Supplementary file 13 — Reporting Summary [file 41467_2021_26203_MOESM13_ESM.pdf]

nature research

Corresponding author(s): Dong Fang

Last updated by author(s): Aug 31, 2021

Reporting Summary

Nature Research wishes to improve the reproducibility of the work that we publish. This form provides structure for consistency and transparency in reporting. For further information on Nature Research policies, see our Editorial Policies and the Editorial Policy Checklist.

Statistics

For all statistical analyses, confirm that the following items are present in the figure legend, table legend, main text, or Methods section.

n/a	Confirmed	
☐	☒	The exact sample size (*n*) for each experimental group/condition, given as a discrete number and unit of measurement
☐	☒	A statement on whether measurements were taken from distinct samples or whether the same sample was measured repeatedly
☐	☒	The statistical test(s) used AND whether they are one- or two-sided<br>*Only common tests should be described solely by name; describe more complex techniques in the Methods section.*
☒	☐	A description of all covariates tested
☒	☐	A description of any assumptions or corrections, such as tests of normality and adjustment for multiple comparisons
☐	☒	A full description of the statistical parameters including central tendency (e.g. means) or other basic estimates (e.g. regression coefficient) AND variation (e.g. standard deviation) or associated estimates of uncertainty (e.g. confidence intervals)
☐	☒	For null hypothesis testing, the test statistic (e.g. *F*, *t*, *r*) with confidence intervals, effect sizes, degrees of freedom and *P* value noted<br>*Give P values as exact values whenever suitable.*
☒	☐	For Bayesian analysis, information on the choice of priors and Markov chain Monte Carlo settings
☒	☐	For hierarchical and complex designs, identification of the appropriate level for tests and full reporting of outcomes
☐	☒	Estimates of effect sizes (e.g. Cohen's *d*, Pearson's *r*), indicating how they were calculated

Our web collection on statistics for biologists contains articles on many of the points above.

Software and code

Policy information about availability of computer code

Data collection	External data were downloaded by sratookit fastq-dump (2. 9.6.1).
Data analysis	Trim Galore (0.6.4), STAR (2.5.4b),featureCounts (2.0.0), Homer (4.11), edgeR (3.28.1), DEseq2 (1.26.0), GATK (4.1.4.0), Bowtie2 (2.3.5.1), MACS2 (2.2.6), BEDTools(2.92.2), deepTools(3.4.3), bedGraphToBigWig (4), corrplot (0.84), pheatmap (1.0.12), VennDiagram (1.6.20), preseq (2.0.3), zUMIs(2.7.1c), Seurat (3.2.1), IGV (2.6.3), ChIPseeker (1.22.1), clusterProfiler (3.14.3), Monocle2 (2.14), STREAM (1.0), cisTopic (0.3.0), WGCNA (1.69), ggalluvial (0.12.3), Custom scripts used in this study are available from https://github.com/Fanglab-zju/scSET-seq

For manuscripts utilizing custom algorithms or software that are central to the research but not yet described in published literature, software must be made available to editors and reviewers. We strongly encourage code deposition in a community repository (e.g. GitHub). See the Nature Research guidelines for submitting code & software for further information.

Data

Policy information about availability of data

All manuscripts must include a data availability statement. This statement should provide the following information, where applicable:

- Accession codes, unique identifiers, or web links for publicly available datasets
- A list of figures that have associated raw data
- A description of any restrictions on data availability

The reference genome used in this study is available in UCSC (http://genome.ucsc.edu) database under mouse reference genome mm9. The raw and processed sequencing data generated in this study have been deposited in the NCBI Gene Expression Omnibus (GEO) database under accession code GSE168637. Encode (https://www.encodeproject.org/) datasets were downloaded with the accession numbers: H3K27me3 (ENCSR059MBO), H3K27me3 input (ENCSR326ULS), H3K4me3 (ENCSR000CGO) and H3K4me3 input (ENCSR095IPH).The other external datasets

were downloaded from NCBI Gene Expression Omnibus (GEO) (http://www.ncbi.nlm.nih.gov/geo/), with the accession numbers: Aebp2 ChIP-seq (GSE83082), Paired-Tag (GSE152020), scATAC-seq (GSE100033), Smart-seq2(GSE151334). 10x scRNA-seq datasets were downloaded from the 10x Genomics website (https://www.10xgenomics.com/).

Field-specific reporting

Please select the one below that is the best fit for your research. If you are not sure, read the appropriate sections before making your selection.

- [x] Life sciences
- [ ] Behavioural & social sciences
- [ ] Ecological, evolutionary & environmental sciences

For a reference copy of the document with all sections, see nature.com/documents/nr-reporting-summary-flat.pdf

Life sciences study design

All studies must disclose on these points even when the disclosure is negative.

Sample size	Sample size was determined based on prior published data from similar experiments (Rosenberg et.al., Science, 2018; Cao et.al., Science, 2018; Preissl et.al., Nat. Neuroscience, 2018)
Data exclusions	Sequencing reads adaptor and low-quality bases were removed by Trim Galore (0.6.4), reads mapped to mitochondria and random chromosomes were also removed. PCR duplicates in epigenetic data were removed by GATK (4.1.4.0).
Replication	All experiments were done for at least twice. scSET-seq for gene expression was conducted through 3 independent experiments. scSET-seq for H3K4me3 and H3K27me3 in Fig. 1 were performed with 5 biological independent experiments respectively. scSET-seq for H3K4me3 and H3K27me3 in Wnt3a-induced asymmetric cell division were conducted from 10 and 6 independent experiments, respectively.
Randomization	Rndomization of reads was selected by bedtools for bioinformatics. Experiments allocation was random.
Blinding	The experiments were not blinded as the identities of histone modification, and the clustering of scSET-seq data were unsupervised.

Reporting for specific materials, systems and methods

We require information from authors about some types of materials, experimental systems and methods used in many studies. Here, indicate whether each material, system or method listed is relevant to your study. If you are not sure if a list item applies to your research, read the appropriate section before selecting a response.

Materials & experimental systems

n/a	Involved in the study
[ ]	[x] Antibodies
[ ]	[x] Eukaryotic cell lines
[x]	[ ] Palaeontology and archaeology
[x]	[ ] Animals and other organisms
[x]	[ ] Human research participants
[x]	[ ] Clinical data
[x]	[ ] Dual use research of concern

Methods

n/a	Involved in the study
[ ]	[x] ChIP-seq
[x]	[ ] Flow cytometry
[x]	[ ] MRI-based neuroimaging

Antibodies

Antibodies used	Rabbit polyclonal anti-Histone H3 (Cat.# ab1791, Abcam), 1:5000 diluted for Western blot;<br>Rabbit polyclonal anti-Histone H3K27me3 (Cat.# 9733, Cell Signaling Technology), 1:1000 diluted for Western blot, 1:50 diluted for SET-seq, 1:200 diluted for scSET-seq;<br>Rabbit polyclonal anti-Histone H3K4me3 (Cat.# ab8580, Abcam), 1:1000 diluted for Western blot, 1:50 diluted for SET-seq, 1:200 diluted for scSET-seq;<br>Rabbit polyclonal anti-Histone H3K9me3 (Cat.# ab8898, Abcam), 1:1000 diluted for Western blot;<br>Rabbit polyclonal anti-Histone H3K27ac (Cat.# ab4729, Abcam), 1:1000 diluted for Western blot;<br>Rabbit monoclonal anti-EZH2 (Cat.# 5246, Clone name D2C9, Cell Signaling Technology), 1:1000 diluted for Western blot;<br>Rabbit monoclonal anti-AEBP2 (Cat.# 14129, Clone name D7C6X, Cell Signaling Technology), 1:1000 diluted for Western blot;<br>Rabbit polyclonal anti-Jarid2 (Cat.# G-2, Novus Biologicals), 1:1000 diluted for Western blot;<br>Peroxidase AffiniPure Goat anti-Rabbit IgG (H + L) (Cat.# 111-035-003, Jackson ImmunoResearch Laboratories), 1:1000 diluted for Western blot.
Validation	H3 were validated in Yuan L et al., Nucleic Acids Res, 2021;<br>H3K27me3 were validated in Chan et al., Genes & Development, 2014;<br>H3K4me3 were validated in Cai Y et al., Nat Commun, 2021;

H3K9me3 were validated in Liang C et al., Cell Res , 2021;
H3K27ac were validated in Wei J et al., Cell, 2021;
EZH2 were validated in Gabriela Vilema-Enríquez et al., J Biol Chem, 2020;
AEBP2 were validated in Siming Chen et al., Mol Cell, 2020;
Jarid2 were validated in Perino M et al., Nat. Genet, 2018.

Eukaryotic cell lines

Policy information about cell lines

Cell line source(s)	Mouse embryonic stem cell ES-E14TG2a from ATCC (CRL-1821)
Authentication	RNA seq
Mycoplasma contamination	All cell lines were negative for mycoplasma contamination.
Commonly misidentified lines (See ICLAC register)	No commonly misidentified cell lines were used in the study.

ChIP-seq

Data deposition

☒ Confirm that both raw and final processed data have been deposited in a public database such as GEO.

☒ Confirm that you have deposited or provided access to graph files (e.g. BED files) for the called peaks.

Data access links
May remain private before publication.

https://www.ncbi.nlm.nih.gov/geo/query/acc.cgi?acc=GSE168637

Files in database submission

H3K27me3_SET-seq_10_cells_Epi_1_rep1.bw,H3K27me3_SET-seq_10_cells_Epi_1_rep1_1.fq.gz,H3K27me3_SET-seq_10_cells_Epi_1_rep1_2.fq.gz,H3K27me3_SET-seq_10_cells_Epi_1_rep2.bw,H3K27me3_SET-seq_10_cells_Epi_1_rep2_1.fq.gz,H3K27me3_SET-seq_10_cells_Epi_1_rep2_2.fq.gz,H3K27me3_SET-seq_100_cells_Epi_1_rep1.bw,H3K27me3_SET-seq_100_cells_Epi_1_rep1_1.fq.gz,H3K27me3_SET-seq_100_cells_Epi_1_rep1_2.fq.gz,H3K27me3_SET-seq_100_cells_Epi_1_rep2.bw,H3K27me3_SET-seq_100_cells_Epi_1_rep2_1.fq.gz,H3K27me3_SET-seq_100_cells_Epi_1_rep2_2.fq.gz,H3K27me3_SET-seq_1000_cells_Epi_1_rep1.bw,H3K27me3_SET-seq_1000_cells_Epi_1_rep1_1.fq.gz,H3K27me3_SET-seq_1000_cells_Epi_1_rep1_2.fq.gz,H3K27me3_SET-seq_1000_cells_Epi_1_rep2.bw,H3K27me3_SET-seq_1000_cells_Epi_1_rep2_1.fq.gz,H3K27me3_SET-seq_1000_cells_Epi_1_rep2_2.fq.gz,H3K27me3_SET-seq_10000_cells_Epi_1_rep1.bw,H3K27me3_SET-seq_10000_cells_Epi_1_rep1_1.fq.gz,H3K27me3_SET-seq_10000_cells_Epi_1_rep1_2.fq.gz,H3K27me3_SET-seq_10000_cells_Epi_1_rep2.bw,H3K27me3_SET-seq_10000_cells_Epi_1_rep2_1.fq.gz,H3K27me3_SET-seq_10000_cells_Epi_1_rep2_2.fq.gz,H3K27me3_SET-seq_10_cells_Epi_2_rep1.bw,H3K27me3_SET-seq_10_cells_Epi_2_rep1_1.fq.gz,H3K27me3_SET-seq_10_cells_Epi_2_rep1_2.fq.gz,H3K27me3_SET-seq_10_cells_Epi_2_rep2.bw,H3K27me3_SET-seq_10_cells_Epi_2_rep2_1.fq.gz,H3K27me3_SET-seq_10_cells_Epi_2_rep2_2.fq.gz,H3K27me3_SET-seq_100_cells_Epi_2_rep1.bw,H3K27me3_SET-seq_100_cells_Epi_2_rep1_1.fq.gz,H3K27me3_SET-seq_100_cells_Epi_2_rep1_2.fq.gz,H3K27me3_SET-seq_100_cells_Epi_2_rep2.bw,H3K27me3_SET-seq_100_cells_Epi_2_rep2_1.fq.gz,H3K27me3_SET-seq_100_cells_Epi_2_rep2_2.fq.gz,H3K27me3_SET-seq_1000_cells_Epi_2_rep1.bw,H3K27me3_SET-seq_1000_cells_Epi_2_rep1_1.fq.gz,H3K27me3_SET-seq_1000_cells_Epi_2_rep1_2.fq.gz,H3K27me3_SET-seq_1000_cells_Epi_2_rep2.bw,H3K27me3_SET-seq_1000_cells_Epi_2_rep2_1.fq.gz,H3K27me3_SET-seq_1000_cells_Epi_2_rep2_2.fq.gz,H3K27me3_SET-seq_10000_cells_Epi_2_rep1.bw,H3K27me3_SET-seq_10000_cells_Epi_2_rep1_1.fq.gz,H3K27me3_SET-seq_10000_cells_Epi_2_rep1_2.fq.gz,H3K27me3_SET-seq_10000_cells_Epi_2_rep2.bw,H3K27me3_SET-seq_10000_cells_Epi_2_rep2_1.fq.gz,H3K27me3_SET-seq_10000_cells_Epi_2_rep2_2.fq.gz,IgG_SET-seq_10000_cells_epi_rep1.bw,IgG_SET-seq_10000_cells_epi_rep1_1.fq.gz,IgG_SET-seq_10000_cells_epi_rep1_2.fq.gz,IgG_SET-seq_10000_cells_epi_rep2.bw,IgG_SET-seq_10000_cells_epi_rep2_1.fq.gz,IgG_SET-seq_10000_cells_epi_rep2_2.fq.gz,vitro-Tn5-25ng/μl_rep1.txt,vitro-Tn5-25ng/μl_rep1_1.fq.gz,vitro-Tn5-25ng/μl_rep1_2.fq.gz,vitro-Tn5-25ng/μl_rep2.txt,vitro-Tn5-25ng/μl_rep2_1.fq.gz,vitro-Tn5-25ng/μl_rep2_2.fq.gz,vitro-Tn5-50ng/μl_rep1.txt,vitro-Tn5-50ng/μl_rep1_1.fq.gz,vitro-Tn5-50ng/μl_rep1_2.fq.gz,vitro-Tn5-50ng/μl_rep2.txt,vitro-Tn5-50ng/μl_rep2_1.fq.gz,vitro-Tn5-50ng/μl_rep2_2.fq.gz,vitro-Tn5-100ng/μl_rep1.txt,vitro-Tn5-100ng/μl_rep1_1.fq.gz,vitro-Tn5-100ng/μl_rep1_2.fq.gz,vitro-Tn5-100ng/μl_rep2.txt,vitro-Tn5-100ng/μl_rep2_1.fq.gz,vitro-Tn5-100ng/μl_rep2_2.fq.gz,vitro-RNA-300ng_rep1.txt,vitro-RNA-300ng_rep1_1.fq.gz,vitro-RNA-300ng_rep1_2.fq.gz,vitro-RNA-300ng_rep2.txt,vitro-RNA-300ng_rep2_1.fq.gz,vitro-RNA-300ng_rep2_2.fq.gz,vitro-RNA-3ng_rep1.txt,vitro-RNA-3ng_rep1_1.fq.gz,vitro-RNA-3ng_rep1_2.fq.gz,vitro-RNA-3ng_rep2.txt,vitro-RNA-3ng_rep2_1.fq.gz,vitro-RNA-3ng_rep2_2.fq.gz,vitro-RNA-30pg_rep1.txt,vitro-RNA-30pg_rep1_1.fq.gz,vitro-RNA-30pg_rep1_2.fq.gz,vitro-RNA-30pg_rep2.txt,vitro-RNA-30pg_rep2_1.fq.gz,vitro-RNA-30pg_rep2_2.fq.gz,vitro-16℃-5mins_rep1.txt,vitro-16℃-5mins_rep1_1.fq.gz,vitro-16℃-5mins_rep1_2.fq.gz,vitro-16℃-5mins_rep2.txt,vitro-16℃-5mins_rep2_1.fq.gz,vitro-16℃-5mins_rep2_2.fq.gz,vitro-16℃-10mins_rep1.txt,vitro-16℃-10mins_rep1_1.fq.gz,vitro-16℃-10mins_rep1_2.fq.gz,vitro-16℃-10mins_rep2.txt,vitro-16℃-10mins_rep2_1.fq.gz,vitro-16℃-10mins_rep2_2.fq.gz,vitro-16℃-15mins_rep1.txt,vitro-16℃-15mins_rep1_1.fq.gz,vitro-16℃-15mins_rep1_2.fq.gz,vitro-16℃-15mins_rep2.txt,vitro-16℃-15mins_rep2_1.fq.gz,vitro-16℃-15mins_rep2_2.fq.gz,vitro-16℃-30mins_rep1.txt,vitro-16℃-30mins_rep1_1.fq.gz,vitro-16℃-30mins_rep1_2.fq.gz,vitro-16℃-30mins_rep2.txt,vitro-16℃-30mins_rep2_1.fq.gz,vitro-16℃-30mins_rep2_2.fq.gz,vitro-37℃-5mins_rep1.txt,vitro-37℃-5mins_rep1_1.fq.gz,vitro-37℃-5mins_rep1_2.fq.gz,vitro-37℃-5mins_rep2.txt,vitro-37℃-5mins_rep2_1.fq.gz,vitro-37℃-5mins_rep2_2.fq.gz,vitro-37℃-10mins_rep1.txt,vitro-37℃-10mins_rep

1_1.fq.gz,vitro-37℃-10mins_rep1_2.fq.gz,vitro-37℃-10mins_rep2.txt,vitro-37℃-10mins_rep2_1.fq.gz,vitro-37℃-10mins_re
p2_2.fq.gz,vitro-37℃-15mins_rep1.txt,vitro-37℃-15mins_rep1_1.fq.gz,vitro-37℃-15mins_rep1_2.fq.gz,vitro-37℃-15mins_r
ep2.txt,vitro-37℃-15mins_rep2_1.fq.gz,vitro-37℃-15mins_rep2_2.fq.gz,vitro-37℃-30mins_rep1.txt,vitro-37℃-30mins_rep
1_1.fq.gz,vitro-37℃-30mins_rep1_2.fq.gz,vitro-37℃-30mins_rep2.txt,vitro-37℃-30mins_rep2_1.fq.gz,vitro-37℃-30mins_re
p2_2.fq.gz,SET-seq_10_cells_Exp_rep1.txt,SET-seq_10_cells_Exp_rep1_1.fq.gz,SET-seq_10_cells_Exp_rep1_2.fq.gz,SET-
seq_10_cells_Exp_rep2.txt,SET-seq_10_cells_Exp_rep2_1.fq.gz,SET-seq_10_cells_Exp_rep2_2.fq.gz,SET-
seq_100_cells_Exp_rep1.txt,SET-seq_100_cells_Exp_rep1_1.fq.gz,SET-seq_100_cells_Exp_rep1_2.fq.gz,SET-
seq_100_cells_Exp_rep2.txt,SET-seq_100_cells_Exp_rep2_1.fq.gz,SET-seq_100_cells_Exp_rep2_2.fq.gz,SET-
seq_1000_cells_Exp_rep1.txt,SET-seq_1000_cells_Exp_rep1_1.fq.gz,SET-seq_1000_cells_Exp_rep1_2.fq.gz,SET-
seq_1000_cells_Exp_rep2.txt,SET-seq_1000_cells_Exp_rep2_1.fq.gz,SET-seq_1000_cells_Exp_rep2_2.fq.gz,SET-
seq_10000_cells_Exp_rep1.txt,SET-seq_10000_cells_Exp_rep1_1.fq.gz,SET-seq_10000_cells_Exp_rep1_2.fq.gz,SET-
seq_10000_cells_Exp_rep2.txt,SET-seq_10000_cells_Exp_rep2_1.fq.gz,SET-
seq_10000_cells_Exp_rep2_2.fq.gz,Ligation_based_RNA-seq_rep1.txt,Ligation_based_RNA-
seq_rep1.fq.gz,Ligation_based_RNA-seq_rep2.txt,Ligation_based_RNA-seq_rep2.fq.gz,H3K4me3_SET-
seq_10^2_cells_Epi_rep1.bw,H3K4me3_SET-seq_10^2_cells_Epi_rep1_1.fq.gz,H3K4me3_SET-
seq_10^2_cells_Epi_rep1_2.fq.gz,H3K4me3_SET-seq_10^2_cells_Epi_rep2.bw,H3K4me3_SET-
seq_10^2_cells_Epi_rep2_1.fq.gz,H3K4me3_SET-seq_10^2_cells_Epi_rep2_2.fq.gz,H3K4me3_SET-
seq_10^3_cells_Epi_rep1.bw,H3K4me3_SET-seq_10^3_cells_Epi_rep1_1.fq.gz,H3K4me3_SET-
seq_10^3_cells_Epi_rep1_2.fq.gz,H3K4me3_SET-seq_10^3_cells_Epi_rep2.bw,H3K4me3_SET-
seq_10^3_cells_Epi_rep2_1.fq.gz,H3K4me3_SET-seq_10^3_cells_Epi_rep2_2.fq.gz,H3K4me3_SET-
seq_10^4_cells_Epi_rep1.bw,H3K4me3_SET-seq_10^4_cells_Epi_rep1_1.fq.gz,H3K4me3_SET-
seq_10^4_cells_Epi_rep1_2.fq.gz,H3K4me3_SET-seq_10^4_cells_Epi_rep2.bw,H3K4me3_SET-
seq_10^4_cells_Epi_rep2_1.fq.gz,H3K4me3_SET-seq_10^4_cells_Epi_rep2_2.fq.gz,H3K4me3_SET-
seq_10^2_cells_Exp_rep1.txt,H3K4me3_SET-seq_10^2_cells_Exp_rep1_1.fq.gz,H3K4me3_SET-
seq_10^2_cells_Exp_rep1_2.fq.gz,H3K4me3_SET-seq_10^2_cells_Exp_rep2.txt,H3K4me3_SET-
seq_10^2_cells_Exp_rep2_1.fq.gz,H3K4me3_SET-seq_10^2_cells_Exp_rep2_2.fq.gz,H3K4me3_SET-
seq_10^3_cells_Exp_rep1.txt,H3K4me3_SET-seq_10^3_cells_Exp_rep1_1.fq.gz,H3K4me3_SET-
seq_10^3_cells_Exp_rep1_2.fq.gz,H3K4me3_SET-seq_10^3_cells_Exp_rep2.txt,H3K4me3_SET-
seq_10^3_cells_Exp_rep2_1.fq.gz,H3K4me3_SET-seq_10^3_cells_Exp_rep2_2.fq.gz,H3K4me3_SET-
seq_10^4_cells_Exp_rep1.txt,H3K4me3_SET-seq_10^4_cells_Exp_rep1_1.fq.gz,H3K4me3_SET-
seq_10^4_cells_Exp_rep1_2.fq.gz,H3K4me3_SET-seq_10^4_cells_Exp_rep2.txt,H3K4me3_SET-
seq_10^4_cells_Exp_rep2_1.fq.gz,H3K4me3_SET-seq_10^4_cells_Exp_rep2_2.fq.gz,H3K27me3_SET-
seq_10^2_cells_Epi_rep1.bw,H3K27me3_SET-seq_10^2_cells_Epi_rep1_1.fq.gz,H3K27me3_SET-
seq_10^2_cells_Epi_rep1_2.fq.gz,H3K27me3_SET-seq_10^2_cells_Epi_rep2.bw,H3K27me3_SET-
seq_10^2_cells_Epi_rep2_1.fq.gz,H3K27me3_SET-seq_10^2_cells_Epi_rep2_2.fq.gz,H3K27me3_SET-
seq_10^3_cells_Epi_rep1.bw,H3K27me3_SET-seq_10^3_cells_Epi_rep1_1.fq.gz,H3K27me3_SET-
seq_10^3_cells_Epi_rep1_2.fq.gz,H3K27me3_SET-seq_10^3_cells_Epi_rep2.bw,H3K27me3_SET-
seq_10^3_cells_Epi_rep2_1.fq.gz,H3K27me3_SET-seq_10^3_cells_Epi_rep2_2.fq.gz,H3K27me3_SET-
seq_10^4_cells_Epi_rep1.bw,H3K27me3_SET-seq_10^4_cells_Epi_rep1_1.fq.gz,H3K27me3_SET-
seq_10^4_cells_Epi_rep1_2.fq.gz,H3K27me3_SET-seq_10^4_cells_Epi_rep2.bw,H3K27me3_SET-
seq_10^4_cells_Epi_rep2_1.fq.gz,H3K27me3_SET-seq_10^4_cells_Epi_rep2_2.fq.gz,H3K27me3_SET-
seq_10^2_cells_Exp_rep1.txt,H3K27me3_SET-seq_10^2_cells_Exp_rep1_1.fq.gz,H3K27me3_SET-
seq_10^2_cells_Exp_rep1_2.fq.gz,H3K27me3_SET-seq_10^2_cells_Exp_rep2.txt,H3K27me3_SET-
seq_10^2_cells_Exp_rep2_1.fq.gz,H3K27me3_SET-seq_10^2_cells_Exp_rep2_2.fq.gz,H3K27me3_SET-
seq_10^3_cells_Exp_rep1.txt,H3K27me3_SET-seq_10^3_cells_Exp_rep1_1.fq.gz,H3K27me3_SET-
seq_10^3_cells_Exp_rep1_2.fq.gz,H3K27me3_SET-seq_10^3_cells_Exp_rep2.txt,H3K27me3_SET-
seq_10^3_cells_Exp_rep2_1.fq.gz,H3K27me3_SET-seq_10^3_cells_Exp_rep2_2.fq.gz,H3K27me3_SET-
seq_10^4_cells_Exp_rep1.txt,H3K27me3_SET-seq_10^4_cells_Exp_rep1_1.fq.gz,H3K27me3_SET-
seq_10^4_cells_Exp_rep1_2.fq.gz,H3K27me3_SET-seq_10^4_cells_Exp_rep2.txt,H3K27me3_SET-
seq_10^4_cells_Exp_rep2_1.fq.gz,H3K27me3_SET-seq_10^4_cells_Exp_rep2_2.fq.gz,Wnt_H3K4me3_SET-
seq_Epi_rep1.bw,Wnt_H3K4me3_SET-seq_Epi_rep1_1.fq.gz,Wnt_H3K4me3_SET-seq_Epi_rep1_2.fq.gz,Wnt_H3K4me3_SET-
seq_Epi_rep2.bw,Wnt_H3K4me3_SET-seq_Epi_rep2_1.fq.gz,Wnt_H3K4me3_SET-
seq_Epi_rep2_2.fq.gz,Wnt_H3K27me3_SET-seq_Epi_rep1.bw,Wnt_H3K27me3_SET-
seq_Epi_rep1_1.fq.gz,Wnt_H3K27me3_SET-seq_Epi_rep1_2.fq.gz,Wnt_H3K27me3_SET-
seq_Epi_rep2.bw,Wnt_H3K27me3_SET-seq_Epi_rep2_1.fq.gz,Wnt_H3K27me3_SET-seq_Epi_rep2_2.fq.gz,H3K4me3_scSET-
seq_Epi.bw,H3K4me3_scSET-seq_Epi_1_1.fq.gz,H3K4me3_scSET-seq_Epi_1_2.fq.gz,H3K4me3_scSET-
seq_Epi.bw,H3K4me3_scSET-seq_Epi_2_1.fq.gz,H3K4me3_scSET-seq_Epi_2_2.fq.gz,H3K4me3_scSET-
seq_Epi.bw,H3K4me3_scSET-seq_Epi_3_1.fq.gz,H3K4me3_scSET-seq_Epi_3_2.fq.gz,H3K4me3_scSET-
seq_Epi.bw,H3K4me3_scSET-seq_Epi_4_1.fq.gz,H3K4me3_scSET-seq_Epi_4_2.fq.gz,H3K4me3_scSET-
seq_Epi.bw,H3K4me3_scSET-seq_Epi_5_1.fq.gz,H3K4me3_scSET-seq_Epi_5_2.fq.gz,H3K27me3_scSET-
seq_Epi.bw,H3K27me3_scSET-seq_Epi_1_1.fq.gz,H3K27me3_scSET-seq_Epi_1_2.fq.gz,H3K27me3_scSET-
seq_Epi.bw,H3K27me3_scSET-seq_Epi_2_1.fq.gz,H3K27me3_scSET-seq_Epi_2_2.fq.gz,H3K27me3_scSET-
seq_Epi.bw,H3K27me3_scSET-seq_Epi_3_1.fq.gz,H3K27me3_scSET-seq_Epi_3_2.fq.gz,H3K27me3_scSET-
seq_Epi.bw,H3K27me3_scSET-seq_Epi_4_1.fq.gz,H3K27me3_scSET-seq_Epi_4_2.fq.gz,H3K27me3_scSET-
seq_Epi.bw,H3K27me3_scSET-seq_Epi_5_1.fq.gz,H3K27me3_scSET-seq_Epi_5_2.fq.gz,Wnt_beads_H3K4me3_scSET-
seq_Epi.bw,Wnt_beads_H3K4me3_scSET-seq_Epi_1_1.fq.gz,Wnt_beads_H3K4me3_scSET-
seq_Epi_1_2.fq.gz,Wnt_beads_H3K4me3_scSET-seq_Epi.bw,Wnt_beads_H3K4me3_scSET-
seq_Epi_2_1.fq.gz,Wnt_beads_H3K4me3_scSET-seq_Epi_2_2.fq.gz,Wnt_beads_H3K4me3_scSET-
seq_Epi.bw,Wnt_beads_H3K4me3_scSET-seq_Epi_3_1.fq.gz,Wnt_beads_H3K4me3_scSET-
seq_Epi_3_2.fq.gz,Wnt_beads_H3K4me3_scSET-seq_Epi.bw,Wnt_beads_H3K4me3_scSET-
seq_Epi_4_1.fq.gz,Wnt_beads_H3K4me3_scSET-seq_Epi_4_2.fq.gz,Wnt_beads_H3K4me3_scSET-
seq_Epi.bw,Wnt_beads_H3K4me3_scSET-seq_Epi_5_1.fq.gz,Wnt_beads_H3K4me3_scSET-
seq_Epi_5_2.fq.gz,Wnt_beads_H3K4me3_scSET-seq_Epi.bw,Wnt_beads_H3K4me3_scSET-
seq_Epi_6_1.fq.gz,Wnt_beads_H3K4me3_scSET-seq_Epi_6_2.fq.gz,Wnt_beads_H3K4me3_scSET-
seq_Exp_counts_matrix.txt,Wnt_beads_H3K4me3_scSET-seq_Exp_1_1.fq.gz,Wnt_beads_H3K4me3_scSET-

seq_Exp_1_2.fq.gz,Wnt_beads_H3K4me3_scSET-seq_Exp_counts_matrix.txt,Wnt_beads_H3K4me3_scSET-seq_Exp_2_1.fq.gz,Wnt_beads_H3K4me3_scSET-seq_Exp_2_2.fq.gz,Wnt_beads_H3K4me3_scSET-seq_Exp_counts_matrix.txt,Wnt_beads_H3K4me3_scSET-seq_Exp_3_1.fq.gz,Wnt_beads_H3K4me3_scSET-seq_Exp_3_2.fq.gz,Wnt_beads_H3K4me3_scSET-seq_Exp_counts_matrix.txt,Wnt_beads_H3K4me3_scSET-seq_Exp_4_1.fq.gz,Wnt_beads_H3K4me3_scSET-seq_Exp_4_2.fq.gz,Wnt_beads_H3K4me3_scSET-seq_Exp_counts_matrix.txt,Wnt_beads_H3K4me3_scSET-seq_Exp_5_1.fq.gz,Wnt_beads_H3K4me3_scSET-seq_Exp_5_2.fq.gz,Wnt_beads_H3K4me3_scSET-seq_Exp_counts_matrix.txt,Wnt_beads_H3K4me3_scSET-seq_Exp_6_1.fq.gz,Wnt_beads_H3K4me3_scSET-seq_Exp_6_2.fq.gz,Wnt_beads_H3K27me3_scSET-seq_Epi.bw,Wnt_beads_H3K27me3_scSET-seq_Epi_1_1.fq.gz,Wnt_beads_H3K27me3_scSET-seq_Epi_1_2.fq.gz,Wnt_beads_H3K27me3_scSET-seq_Epi.bw,Wnt_beads_H3K27me3_scSET-seq_Epi_2_1.fq.gz,Wnt_beads_H3K27me3_scSET-seq_Epi_2_2.fq.gz,Wnt_beads_H3K27me3_scSET-seq_Epi.bw,Wnt_beads_H3K27me3_scSET-seq_Epi_3_1.fq.gz,Wnt_beads_H3K27me3_scSET-seq_Epi_3_2.fq.gz,Wnt_beads_H3K27me3_scSET-seq_Epi.bw,Wnt_beads_H3K27me3_scSET-seq_Epi_4_1.fq.gz,Wnt_beads_H3K27me3_scSET-seq_Epi_4_2.fq.gz,Wnt_beads_H3K27me3_scSET-seq_Epi.bw,Wnt_beads_H3K27me3_scSET-seq_Epi_5_1.fq.gz,Wnt_beads_H3K27me3_scSET-seq_Epi_5_2.fq.gz,Wnt_beads_H3K27me3_scSET-seq_Epi.bw,Wnt_beads_H3K27me3_scSET-seq_Epi_6_1.fq.gz,Wnt_beads_H3K27me3_scSET-seq_Epi_6_2.fq.gz,Wnt_beads_H3K27me3_scSET-seq_Epi.bw,Wnt_beads_H3K27me3_scSET-seq_Epi_7_1.fq.gz,Wnt_beads_H3K27me3_scSET-seq_Epi_7_2.fq.gz,Wnt_beads_H3K27me3_scSET-seq_Epi.bw,Wnt_beads_H3K27me3_scSET-seq_Epi_8_1.fq.gz,Wnt_beads_H3K27me3_scSET-seq_Epi_8_2.fq.gz,Wnt_beads_H3K27me3_scSET-seq_Epi.bw,Wnt_beads_H3K27me3_scSET-seq_Epi_9_1.fq.gz,Wnt_beads_H3K27me3_scSET-seq_Epi_9_2.fq.gz,Wnt_beads_H3K27me3_scSET-seq_Epi.bw,Wnt_beads_H3K27me3_scSET-seq_Epi_10_1.fq.gz,Wnt_beads_H3K27me3_scSET-seq_Epi_10_2.fq.gz,Wnt_beads_H3K27me3_scSET-seq_Exp_counts_matrix.txt,Wnt_beads_H3K27me3_scSET-seq_Exp_1_1.fq.gz,Wnt_beads_H3K27me3_scSET-seq_Exp_1_2.fq.gz,Wnt_beads_H3K27me3_scSET-seq_Exp_counts_matrix.txt,Wnt_beads_H3K27me3_scSET-seq_Exp_2_1.fq.gz,Wnt_beads_H3K27me3_scSET-seq_Exp_2_2.fq.gz,Wnt_beads_H3K27me3_scSET-seq_Exp_counts_matrix.txt,Wnt_beads_H3K27me3_scSET-seq_Exp_3_1.fq.gz,Wnt_beads_H3K27me3_scSET-seq_Exp_3_2.fq.gz,Wnt_beads_H3K27me3_scSET-seq_Exp_counts_matrix.txt,Wnt_beads_H3K27me3_scSET-seq_Exp_4_1.fq.gz,Wnt_beads_H3K27me3_scSET-seq_Exp_4_2.fq.gz,Wnt_beads_H3K27me3_scSET-seq_Exp_counts_matrix.txt,Wnt_beads_H3K27me3_scSET-seq_Exp_5_1.fq.gz,Wnt_beads_H3K27me3_scSET-seq_Exp_5_2.fq.gz,Wnt_beads_H3K27me3_scSET-seq_Exp_counts_matrix.txt,Wnt_beads_H3K27me3_scSET-seq_Exp_6_1.fq.gz,Wnt_beads_H3K27me3_scSET-seq_Exp_6_2.fq.gz,Wnt_beads_H3K27me3_scSET-seq_Exp_counts_matrix.txt,Wnt_beads_H3K27me3_scSET-seq_Exp_7_1.fq.gz,Wnt_beads_H3K27me3_scSET-seq_Exp_7_2.fq.gz,Wnt_beads_H3K27me3_scSET-seq_Exp_counts_matrix.txt,Wnt_beads_H3K27me3_scSET-seq_Exp_8_1.fq.gz,Wnt_beads_H3K27me3_scSET-seq_Exp_8_2.fq.gz,Wnt_beads_H3K27me3_scSET-seq_Exp_counts_matrix.txt,Wnt_beads_H3K27me3_scSET-seq_Exp_9_1.fq.gz,Wnt_beads_H3K27me3_scSET-seq_Exp_9_2.fq.gz,Wnt_beads_H3K27me3_scSET-seq_Exp_counts_matrix.txt,Wnt_beads_H3K27me3_scSET-seq_Exp_10_1.fq.gz,Wnt_beads_H3K27me3_scSET-seq_Exp_10_2.fq.gz,H3K27me3_Epi_Aebp2_KO1_rep1.bw,H3K27me3_Epi_Aebp2_KO1_rep1_1.fq.gz,H3K27me3_Epi_Aebp2_KO1_rep1_2.fq.gz,H3K27me3_Epi_Aebp2_KO1_rep2.bw,H3K27me3_Epi_Aebp2_KO1_rep2_1.fq.gz,H3K27me3_Epi_Aebp2_KO1_rep2_2.fq.gz,H3K27me3_Epi_Aebp2_KO2_rep1.bw,H3K27me3_Epi_Aebp2_KO2_rep1_1.fq.gz,H3K27me3_Epi_Aebp2_KO2_rep1_2.fq.gz,H3K27me3_Epi_Aebp2_KO2_rep2.bw,H3K27me3_Epi_Aebp2_KO2_rep2_1.fq.gz,H3K27me3_Epi_Aebp2_KO2_rep2_2.fq.gz,H3K27me3_Exp_Aebp2_KO1_rep1.txt,H3K27me3_Exp_Aebp2_KO1_rep1_1.fq.gz,H3K27me3_Exp_Aebp2_KO1_rep1_2.fq.gz,H3K27me3_Exp_Aebp2_KO1_rep2.txt,H3K27me3_Exp_Aebp2_KO1_rep2_1.fq.gz,H3K27me3_Exp_Aebp2_KO1_rep2_2.fq.gz,H3K27me3_Exp_Aebp2_KO2_rep1.txt,H3K27me3_Exp_Aebp2_KO2_rep1_1.fq.gz,H3K27me3_Exp_Aebp2_KO2_rep1_2.fq.gz,H3K27me3_Exp_Aebp2_KO2_rep2.txt,H3K27me3_Exp_Aebp2_KO2_rep2_1.fq.gz,H3K27me3_Exp_Aebp2_KO2_rep2_2.fq.gz

Genome browser session
(e.g. UCSC)

no longer applicable

Methodology

Replicates

Each sequencing was repeated at least twice.

Sequencing depth

The numbers of reads and unique mapped reads is summarized in Supplementary Dataset 7.
All reads are sequenced as 150bp with paired-end .

Antibodies

Rabbit polyclonal anti-Histone H3K27me3 (Cat# 9733, Cell Singaling Technology); Rabbit polyclonal anti-Histone H3K4me3 (Cat# ab8580, Abcam)

Peak calling parameters

For H3K27me3
macs2 callpeak --broad -g mm –nomodel
For H3K4me3
macs2 callpeak -g mm -B --nomodel
Index files used for mapping is mm9 genome

Data quality

Peak qualities were provided as Supplementary Dataset 8.

Software

Trim Galore (0.6.4), Homer (4.11),GATK (4.1.4.0), Bowtie2 (2.3.5.1), MACS2 (2.2.6), BEDTools (2.92.2), deepTools(version 3.4.3), bedGraphToBigWig (4), VennDiagram (1.6.20), preseq (2.0.3), IGV (2.6.3), ChIPseeker(1.22.1)
